# Key Programme Science lessons from an HIV prevention ‘Learning Site’ for sex workers in Mombasa, Kenya

**DOI:** 10.1136/sextrans-2017-053228

**Published:** 2017-12-14

**Authors:** Leigh M McClarty, Parinita Bhattacharjee, Shajy Isac, Faran Emmanuel, Japheth Kioko, Margaret Njiraini, Peter Gichangi, Clifford Duncan Okoth, Janet Musimbi-Mbole, James F Blanchard, Stephen Moses, Helgar Muysyoki, Marissa L Becker

**Affiliations:** 1 Department of Community Health Sciences, Centre for Global Public Health, University of Manitoba, Winnipeg, Manitoba, Canada; 2 National AIDS and STI Control Programme, Ministry of Health, Government of Kenya, Nairobi, Kenya; 3 Partners for Health and Development in Africa, Nairobi, Kenya; 4 International Centre for Reproductive Health Kenya, Mombasa, Kenya; 5 Community Advisory Board, Learning Site, Mombasa, Kenya

**Keywords:** Africa, prevention, commercial Sex, programme science

## Abstract

**Objectives:**

In 2013, Kenya’s National AIDS and STI Control Programme established a Learning Site (LS) in Mombasa County to support and strengthen capacity for HIV prevention programming within organisations working with sex workers. A defining feature of LS was the use of a Programme Science approach throughout its development and implementation. We provide an overview of the key components of LS, present findings from 23 months of programme monitoring data, and highlight key Programme Science lessons from its implementation and monitoring.

**Methods:**

Routine monitoring data collected from September 2013 through July 2015 are presented. Individual-level service utilisation data were collected monthly and indicators of interest were analysed over time to illustrate trends in enrolment, programme coverage and service utilisation among sex workers in Mombasa County.

**Results:**

Over the monitoring period, outreach programme enrolment occurred rapidly; condom distribution targets were met consistently; rates of STI screening remained high and diagnoses declined; and reporting of and response to violent incidents increased. At the same time, enrolment in LS clinics was relatively low among female sex workers, and HIV testing at LS was low among both female and male sex workers.

**Conclusion:**

Lessons learnt from operationalising the Programme Science framework through the Mombasa LS can inform the development and implementation of similar LS in different geographical and epidemiological contexts. Importantly, meaningful involvement of sex workers in the design, implementation and monitoring processes ensures that overall programme performance is optimised in the context of local, ‘on-the-ground’ realities. Additionally, learnings from LS highlight the importance of introducing enhanced monitoring and evaluations systems into complex programmes to better understand and explain programme dynamics over time.

## Introduction

Kenya has a generalised HIV epidemic, with an estimated adult prevalence of 5.9%^1^ and substantial epidemiological heterogeneity. HIV incidence and prevalence is highest among members of key populations who are considered to be disproportionately vulnerable to infection.^2^ In Kenya, key populations contribute an estimated 30% of new HIV infections annually, with female sex workers contributing approximately 14% and men who have sex with men—including male sex workers—contributing another 15% in 2014.^3^ Geographical heterogeneity in HIV incidence and prevalence also exists across the country;^1 3 4^ Mombasa County has the seventh highest HIV prevalence in Kenya, estimated to be 7.5% in 2015.^1^

In 2013, Kenya’s National AIDS and STI Control Programme (NASCOP) established a Learning Site (LS) in two subcounties of Mombasa County.^5^ The LS aimed to support and strengthen capacity for HIV and other STI prevention and care programming in non-governmental and community-based organisations (CBOs) working with sex workers. A technical support unit set up by the University of Manitoba’s Centre for Global Public Health provided support to NASCOP, while implementation occurred in partnership with the International Centre for Reproductive Health Kenya and several Mombasa-based, sex worker-led CBOs.

The objective of LS was to develop and implement a model HIV prevention and care programme specifically tailored to accommodate the needs of female and male sex workers in line with national guidelines.^3 6^ A defining feature of LS was its use of a Programme Science approach for programme development and implementation. Programme Science systematically applies scientific theory, methods and findings to address context-specific complexities and improve the planning, implementation and evaluation of programmes.^7 8^ In the context of LS, this meant that: (1) service delivery was prioritised for geographical areas and key populations disproportionately burdened by HIV using epidemiological mapping exercises prior to programme implementation; (2) relevant, evidence-based, sex worker (peer)-led interventions were developed in consultation with key populations; (3) optimal service delivery was established through microplanning; and (4) programme monitoring and responsive tactical and strategic adaptations occurred, at the individual and programme levels, throughout the programme’s life span.

This article provides an overview of the key components of the Mombasa LS, presents findings from programme monitoring data, and highlights key Programme Science lessons that can inform the design and implementation of other HIV prevention programmes for sex workers.

## Methods

### Data sources and analyses

Routine programme monitoring data, collected from September 2013 through July 2015, were analysed. On enrolment with LS, sex workers were provided with information about outreach and sexual health services offered by the programme and assigned unique identification codes to maintain individual anonymity. These codes were consistent for an individual for the duration of the programme and were used to monitor utilisation of services provided by LS. Individual-level service utilisation data were collected regularly, including indicators for enrolment and contact with the outreach programme and utilisation of clinical services and violence/crisis response interventions. Data were entered into Microsoft Access monthly, then exported to SPSS V.20 and Microsoft Excel for analyses. Indicators of interest were analysed over time—either monthly or quarterly, depending on how targets were set—to illustrate trends in enrolment, programme coverage and service utilisation. Where applicable, previously derived size estimates for local female and male sex worker populations were used as denominators for programme outputs.^5^ For the purposes of LS, male sex workers were defined as men who provide sexual services to other men in exchange for cash or other resources.

### The Mombasa LS

The LS included behavioural, biomedical and structural intervention components, which together comprised a comprehensive combination HIV prevention programme,^9–11^ the preferred model endorsed by NASCOP.^3^ A community advisory board, whose members represented 13 Mombasa-based CBOs, was a key component of LS. The community advisory board provided feedback and guidance, and ensured that LS remained accountable and relevant to sex worker communities.

Briefly, the behavioural intervention used a peer-based model in which peer educators (who were members of local sex worker communities) were responsible for enrolling sex workers in an outreach programme, developing personalised outreach plans through hotspot-based microplanning strategies,^12^ and distributing condoms and lubricants. ‘Hotspots’ were defined as any physical location where sex workers met and/or engaged in sexual intercourse with partners/clients. Due to constrained resources, lubricants were only actively distributed to male sex workers, but were available to female sex workers on request. Through microplanning, peer educators recorded information from outreach activities in weekly tracking sheets, reviewed the data in monthly meetings with other peer educators, then, when necessary, implemented modifications to outreach plans based on microplanning data. The biomedical intervention was clinic-based and located at a sex worker hotspot. The clinic offered free, comprehensive sexual health services exclusively to sex workers in a safe space. Available services included quarterly STI screening and treatment,^13^ quarterly HIV testing and referrals to government-approved antiretroviral therapy centres. Clinic hours were adjusted to accommodate sex workers’ schedules, and mobile clinics were periodically conducted in hot spots to reach sex workers who could not reach the stationary clinic. Finally, the LS addressed experiences of violence within the sex worker community through structural interventions.^14 15^ The first component was a drop-in centre that provided a safe and supportive space for sex workers to socialise and rest. The second component was a 24 hours hotline through which sex workers could report violent incidents, gain immediate access to the LS crisis management team, receive legal advice from peers trained as paralegals and be referred to appropriate services.

A full description of the intervention components of the Mombasa LS is included in the online supplementary appendix 1.

10.1136/sextrans-2017-053228.supp1 Supplementary file 1

### Ethical considerations

All data collected through routine programme monitoring were anonymous and de-identified.

## Results

### Outreach programme coverage

Based on mapping exercises preceding LS implementation,^5^ an estimated 5809 female sex workers and 600 male sex workers worked within the targeted subcounties. As per national guidelines,^16^ LS aimed to enrol 80% of the estimated sex worker population into its outreach programme and have monthly contact with all enrolled.

**Figure 1:**
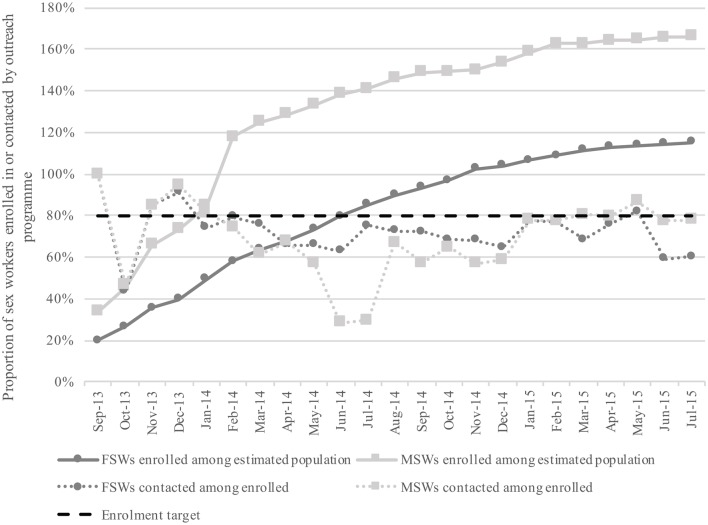
Cumulative monthly enrolment for female and male sex workers as a proportion of estimated population sizes, and proportion of enrolled sex workers contacted by programme per month, September 2013 through July 2015. FSWs, female sex workers; MSWs, male sex workers.

Cumulative enrolment in the outreach programme increased steadily over the 23-month monitoring period, with 80% of the estimated female and male sex worker populations being reached after 10 months and 5 months, respectively (figure 1). The number of sex workers enrolled exceeded initial size estimates. The proportion of LS-enrolled sex workers who were contacted by the programme varied by month, but largely remained between 50% and 80%.

### Condom and personal lubricant distribution

**Figure 2:**
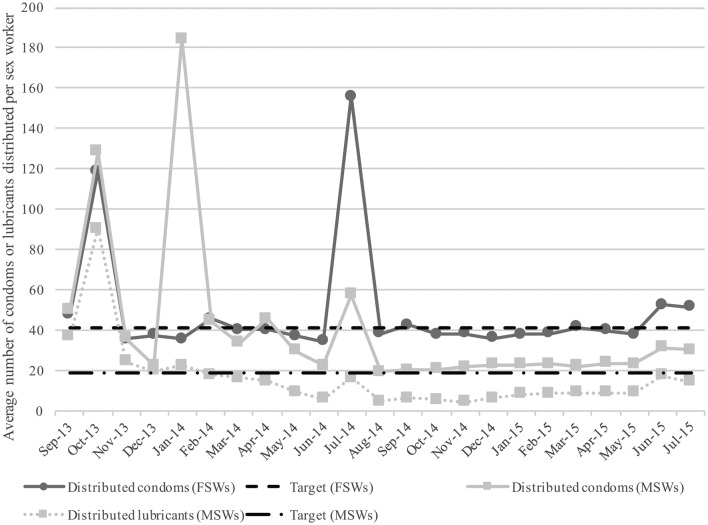
Average number of condoms distributed per contacted female sex worker, and condoms and lubricants distributed per contacted male sex worker per month, September 2013 through July 2015. Monthly distribution targets set at 41.0 and 19.0 per female and male sex worker, respectively. FSWs, female sex workers; MSWs, male sex workers.

The average number of condoms distributed to female sex workers each month remained relatively constant over the monitoring period, although during a few months, distribution far exceeded anticipated need (figure 2). Condom and lubricant distribution to male sex workers varied in the first half of the monitoring period, but stabilised by the 12th month. Distribution of personal lubricants among male sex workers was consistently lower than condom distribution (figure 2). Lubricant distribution to female sex workers was not monitored.^16^

### Health service utilisation

While the crude number of sex workers registered with the LS clinic increased over 23 months, the proportion of all sex workers who were enrolled in the clinic, and the proportion contacted monthly, remained relatively stable (online supplementary figure 1). In the first month of monitoring, 48.0% (n=522) of outreach-enrolled female sex workers had also registered with the clinic, and by the 23rd month of monitoring, this increased to 55.8% (n=3736). The proportion of outreach-enrolled male sex workers who were also enrolled with the clinic decreased from 98.5% (n=198) in the first month to 86.0% (n=856) in the 23rd month.

10.1136/sextrans-2017-053228.supp2 Supplementary file 2

The proportion of clinic-enrolled sex workers who attended the clinic quarterly varied, but remained below the 60% target (online supplementary figure 2). Quarterly trends in attendance were similar for both female sex workers and male sex workers.

**Figure 3:**
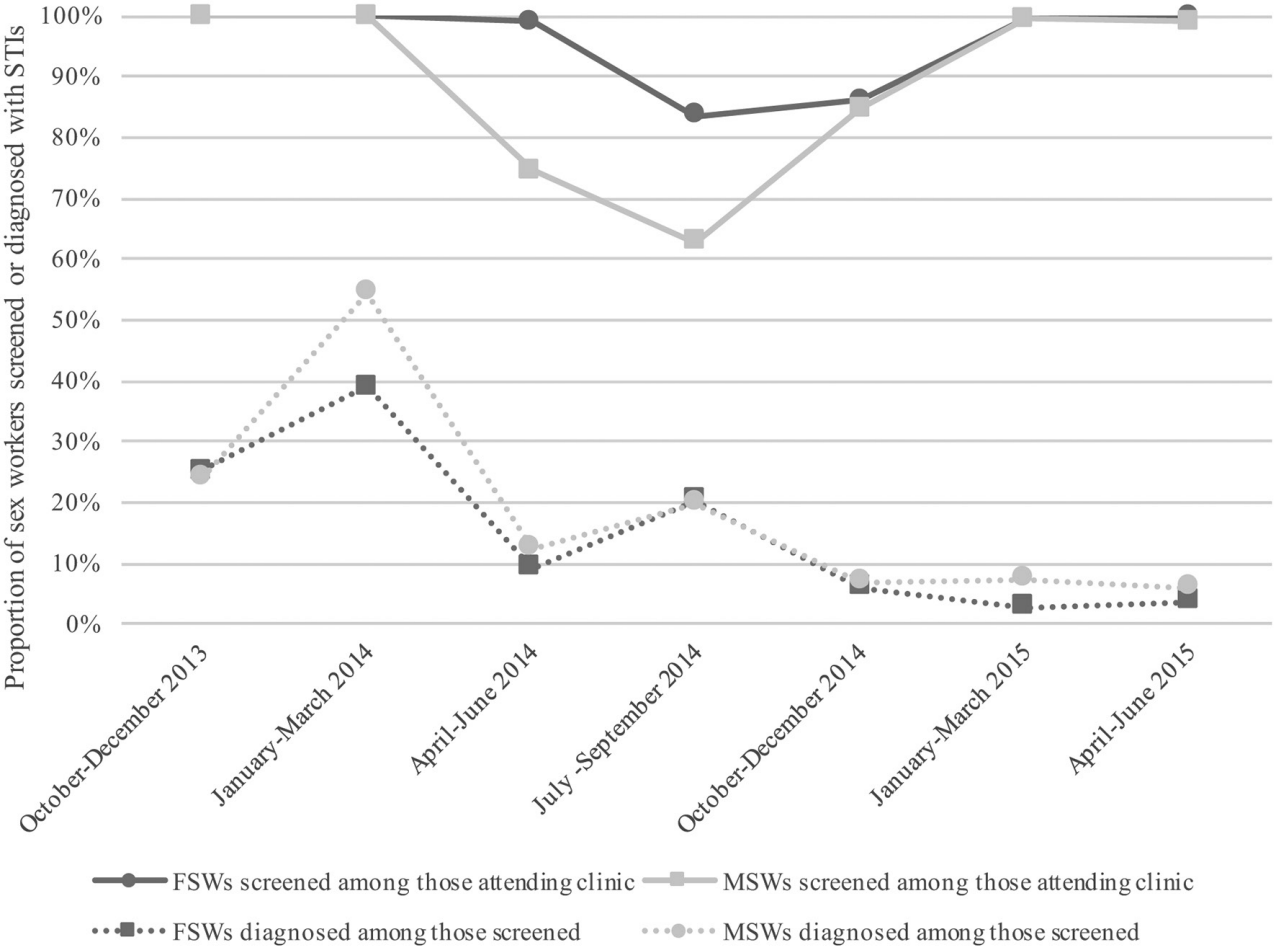
Proportion of sex workers screened for STIs quarterly among clinic attendees, and proportion of sex workers diagnosed with STIs among those screened per quarter, October 2013 through June 2015. FSWs, female sex workers; MSWs, male sex workers.

Among sex workers attending clinic, over 60% were consistently screened for STIs, and by the end of the monitoring period, 100% were being screened, meeting the established target (figure 3). During the same period, rates of STI diagnoses among screened clinic attendees decreased from 25.1% to 3.7% for female sex workers, and from 24.0% to 5.9% among male sex workers (figure 3).

**Figure 4:**
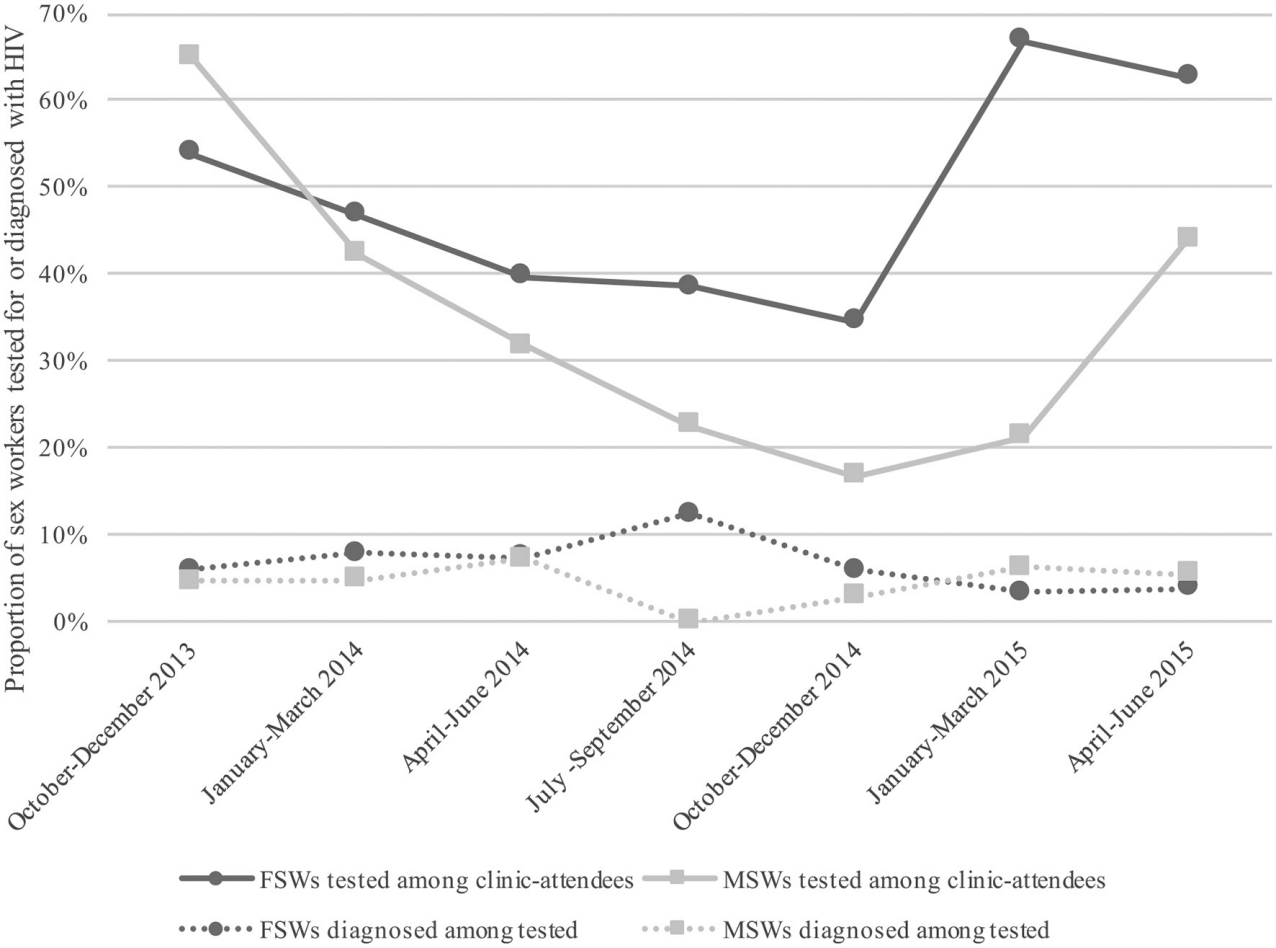
Proportion of sex workers tested for HIV quarterly among clinic attendees, and proportion diagnosed with HIV among those tested per quarter, October 2013 through June 2015. FSWs, female sex workers; MSWs, male sex workers.

The proportion of clinic attendees who were tested for HIV decreased over the first five quarters of the monitoring period, then began to increase in the final two quarters (figure 4). Among those tested for HIV, the proportion of positive diagnoses remained between 3% and 8% in all but one quarter. HIV diagnoses among female sex workers decreased between the first and last quarters, from 5.9% to 3.8%, while the proportion of positive diagnoses among male sex workers increased slightly, from 4.6% to 5.6% (figure 4).

### Violent incident and crisis reporting and response

Violent incidents reported through the 24 hours crisis telephone line peaked in months 13 and 14 (online supplementary figure 3). By the fifth month, each reported incident was addressed within 24 hours, per LS protocol. For most months during the monitoring period, a greater number of violent incidents were reported by female sex workers than male sex workers.

## Discussion

Several positive findings emerged during and following the implementation of LS. Outreach programme enrolment occurred rapidly; condom distribution targets were met consistently; rates of STI screening were high, and remained high; and STI diagnoses declined. Additionally, reporting of and subsequent response to violent incidents among sex workers substantially increased within 5 months of introducing the crisis response system.

Nevertheless, LS was not without challenges, many of which presented important learning opportunities. Programme monitoring data showed relatively low rates of clinic attendance among outreach-enrolled female sex workers, which may be partly explained by the existence of other clinics in Mombasa offering similar services concurrently with LS. However, attendance among outreach-enrolled male sex workers was relatively high, suggesting that LS filled an important service gap in Mombasa. Recent findings from polling booth surveys (PBS) conducted by NASCOP^14 17^ showed a significant increase in the proportion of both female sex workers and men who have sex with men (including male sex workers) in Mombasa who visited intervention clinics between 2014 and 2015. In 2014, 29% of female sex workers reported visiting the clinics, increasing to 75% in 2015 (P<0.001), while 47% of men who have sex with men attended a clinic in 2014, compared with 57% in 2015 (P<0.05).^17^ Analysing monitoring data in the context of PBS findings highlights the plausibility of LS contributing to an overall increase in accessibility and utilisation of clinical services for sex workers at the population level. However, the 60% clinic attendance target was not achieved during the monitoring period. This suggests that while initial engagement is important for linkage to care, more diverse community-based service delivery options that are not dependent on facility-based service delivery (eg, self-testing, peer-testing, point-of-care testing)^18–21^ should be considered.

Another gap identified was a steady decrease in the proportion of clinic attendees receiving HIV tests during the first five quarters of the monitoring period, despite population-based PBS data from Mombasa showing a significantly greater proportion of sex workers reporting HIV testing within the last 3 months in 2015 compared with 2014 (94% versus 71%, respectively; P<0.0001).^17^ As previously noted, other testing centres exist in Mombasa County, so it is conceivable that sex workers were receiving HIV testing elsewhere.

### Key Programme Science lessons

We now highlight three key Programme Science lessons learnt from LS pertaining to planning, implementing and monitoring comprehensive HIV prevention programmes for sex workers.

First, LS demonstrated that rapid programme enrolment is achievable among key populations through Programme Science principles, including strategic planning and allocation of resources using knowledge generated from mapping and effective engagement with peers throughout (figure 1). Mapping has been demonstrated as an important strategy for developing key population size estimates and informing programmes about where to focus interventions across a variety of settings.^22 23^ In Mombasa, mapping results provided critical information about the number and location of hotspots with the highest densities of sex workers in which to prioritise and focus enhanced enrolment efforts. This included a focus on reaching sex workers who had not yet been contacted by the programme with directed outreach activities. Engaging sex workers as peer educators helped to foster and maintain good rapport with the community, which greatly facilitated rapid programme enrolment and service uptake. Additionally, by using peer-based enrolment strategies, LS was able to reach an even larger number of sex workers than initially enumerated (figure 1). These findings support previous research^23^ indicating that mapping should be performed iteratively over a programme’s life span to capture changes in the size and geographical distribution of a target population over time, to monitor hotspot turnover, and to better understand mobility among sex worker populations.

Second, in accordance with the Programme Science framework, LS illustrated benefits of using a monitoring and evaluation (M&E) strategy involving (at least) two ‘tiers’ of monitoring to strengthen a programme’s responsiveness to the dynamic context in which it exists. Within a first tier, microplanning also functions as a ‘micromonitoring’ strategy that allows front-line service providers (for example, peer educators) to: (1) understand the needs of local sex workers; (2) establish programme targets based on need; and (3) assess whether needs and/or programme targets are being met. In this way, micromonitoring becomes a peer-led process that actively engages front-line service providers in programme implementation by monitoring service delivery at the individual level, through frequent review and analysis of programme indicators. Micromonitoring lends nimbleness to a programme that allows for quick responses to divergences from established targets, contributing to overall programme efficiency. A second tier, ‘macromonitoring’, can be conceptualised as a programme-level process performed by an M&E team that identifies suboptimal or undesirable trends in overall programme performance at the programme-population level. Macromonitoring involves gap analyses of programme outputs on a quarterly or semiannual basis that can help programme implementers make informed decisions about whether and how to introduce appropriate mid-course corrections to accommodate dynamic and context-specific needs of the target population(s). For example, when analyses of monitoring data revealed that HIV testing was declining (figure 4), a programme-level response resulted in LS offering outreach testing clinics to increase accessibility at hotspots outside of the LS clinic. Furthermore, it is beneficial to analyse macromonitoring data in relation to population-based data, such as those gathered by PBS, to contextualise programme outputs within population-level trends.

The third Programme Science lesson was that while microplanning can increase efficiency of service delivery within targeted HIV prevention programmes, improved M&E protocols for process documentation during microplanning activities could further strengthen programming. Systematically documenting modifications made to peer-based service delivery strategies would also help in explaining how decisions made based on individual-level data influence trends in service provision and utilisation at the programme-population level. In the context of LS, microplanning enabled peer educators to collect and review individual-level service delivery data on a monthly basis. Whenever discrepancies were noted between established condom/lubricant distribution targets and the number actually distributed, a plan was collectively devised among peer educators to address service delivery gaps. While this strategy seemed to work well for condom distribution—as demonstrated by the rapid response to correct overdistribution in months 5 and 11 (figure 2)—distribution of personal lubricants to male sex workers was consistently below the established target. This is despite national guidelines indicating that condoms and personal lubricants should be distributed together as a package to male sex workers.^16^ From existing LS monitoring data, it is impossible to decipher whether this discrepancy was due to suboptimal implementation or an intentional response to a change in service needs. A comprehensive, peer-informed system to document and track decision-making processes around modifying implementation strategies would complement existing M&E efforts by enhancing interpretation of programme monitoring data and explaining why discrepancies between outputs and their targets might persist over time.

### Limitations to programme monitoring data

Analyses using programme monitoring data are inherently limited; the data presented in this paper are not meant to serve as a formal impact evaluation of LS. Instead, we aimed to examine the application of a Programme Science framework in the context of a comprehensive HIV/STI prevention programme for key populations.

Strategies to enhance the programme’s M&E should include the systematic and thoughtful incorporation of qualitative methodologies into monitoring protocols. For example, standard quantitative indicators could be enhanced and contextualised through qualitative inquiry with programme staff about their experiences and decision-making processes around programme implementation. A clearer picture of the dynamic nature of a programme may begin to emerge, allowing for a more holistic understanding of observed trends and providing important programme knowledge. Furthermore, having qualitative data readily available within a programme’s M&E framework would importantly facilitate meaningful evaluations of complex intervention programmes, like the Mombasa LS.^24^

Finally, while micromonitoring and macromonitoring data are useful to observe trends and patterns in programme outcomes, neither are sufficient to understand why or how these trends and patterns occur. For the Mombasa LS specifically, relying solely on monitoring data to understand processes related to programme change highlighted an important limitation in the programme’s M&E framework. There is a need for comprehensive systems of process documentation to track decisions and record rationale for mid-course changes and modifications made to components of complexprogrammes over time.

## Conclusion

Operationalisation of the Programme Science framework in the Mombasa LS presents several learning opportunities for HIV prevention programmes and implementing organisations. One theme that emerged consistently was the importance of meaningfully involving sex workers in programme design, implementation and monitoring to ensure that overall programme performance is optimised in the context of local, ‘on-the-ground’ realities. At the same time, the programme itself can act as a space in which crucial community collectivisation emerges and thrives. Meaningful community involvement also ensures that sex workers have control and ownership of the programme, which can contribute significantly to overall empowerment within their communities.^25^ Furthermore, by examining LS through a Programme Science lens, it became clear that standard M&E systems could be substantially improved to produce data that are conducive to more meaningful analyses and nuanced interpretations of how programme implementation can affect programme outputs.

**Key messages**

- Operationalising Programme  Science in the context of the Mombasa Learning Site facilitated rapid programme enrolment, efficient service delivery and responsive programme monitoring strategies.
- Meaningfully involving community members in the design, implementation and monitoring processes ensures that overall programme performance is optimised in the context of local, ‘on-the-ground’ realities.
- It is beneficial to use monitoring data to contextualise programme outputs within population-level trends in HIV/STI incidence and treatment and prevention service utilisation and uptake.
- Enhanced monitoring and evaluation systems incorporating qualitative methodologies and focusing on process documentation may lend themselves to more comprehensive understandings of programme dynamics over time.
