# Supplementary material for: Key Programme Science lessons from an HIV prevention ‘Learning Site’ for sex workers in Mombasa, Kenya
Source: Sex Transm Infect. 2017 Dec 14;94(5):346–52. doi: 10.1136/sextrans-2017-053228 (PMC6204943; doi:10.1136/sextrans-2017-053228)
Supplement: Supplementary file 1 [file sextrans-2017-053228supp001.docx]

# WEB APPENDIX 1

## Description of intervention components of the Mombasa Learning Site

The behavioural intervention used a peer-based outreach model in which peer educators (who were members of local sex worker communities) were responsible for: enrolling sex workers in the outreach programme; building good rapport with those enrolled; providing information about HIV, STIs, and locally available testing and treatment resources; distributing condoms and lubricants; discussing feasible condom negotiation strategies; and demonstrating proper condom use. Enrolment efforts and outreach activities occurred simultaneously in hotspots that had been identified in an earlier mapping exercise,^1^ and were re-validated by the LS outreach team. To optimise outreach efforts, hotspots at which the greatest number of sex workers operated were prioritised. Peer educators employed hotspot-based microplanning strategies^2^ to develop personalised outreach plans using peer planning tools that considered a sex worker’s age, duration in sex work, client volume, and experience with violence. Condoms were regularly distributed to both female and male sex workers; however, due to constrained resources, lubricants were only actively distributed to male sex workers, but were made available to female sex workers upon request. Peer planning tools helped estimate each sex worker’s average monthly condom requirement, optimise condom/lubricant distribution according to need, and predict total condom/lubricant requirements for the programme. Through the process of microplanning,^2^ peer educators recorded information from interactions with sex workers during outreach in weekly tracking sheets, then reviewed the data in monthly meetings with other peer educators and outreach workers. Based on these reviews, plans for the following month were made and, if indicated, revisions or modifications to outreach plans were incorporated.

The biomedical intervention component of the LS was a clinic, strategically located behind a busy nightclub that was a validated hotspot, which offered free, comprehensive sexual health services exclusively to sex workers in a welcoming and safe space. Quarterly STI screening was performed by a clinician at each clinic visit based on a standardised syndromic management chart, and treatment was offered according to national guidelines.^3^ HIV testing services were also offered quarterly through the clinic, and anyone who tested HIV-positive was referred to a government-approved antiretroviral therapy (ART) centre, invited to take part in LS-organised peer support groups, and followed up quarterly by LS clinicians. To facilitate linkage to HIV treatment, and to reduce economic barriers to accessing these services, the LS arranged and covered expenses for accompanied referrals to first visits to ART centres for routine HIV blood work and antiretroviral medications. The actual provision of medication through ART centres, which was under the purview of the Government of Kenya. To increase accessibility of clinical services, clinic hours were extended to include evenings and weekends, and mobile clinics were periodically conducted in hotspots to reach sex workers who might not attend the stationary clinic.

Finally, using a selection of structural approaches, the LS aimed to address experiences of violence and foster solidarity within the sex worker community.^4 5^ The first intervention was a drop-in centre (DIC), created as a community-run “safe space”—a strategy shown to be a valuable component of community-based HIV prevention programmes for other marginalised groups.^6^ The DIC included a shower, sleeping accommodations, common areas for socialising, and computer facilities. Activities within the DIC were developed by sex workers who used the space, and were centred on creating and maintaining a respectful and supportive environment for all users. The second structural intervention was a crisis response system, which included a 24-hour emergency telephone line. Sex workers frequently experience on-the-job violence at the hands of clients, intimate partners, state actors, and other authority figures—including physical/verbal abuse, sexual assault, discrimination, and arrest.^7-9^ Through the crisis response system, sex workers could gain immediate access to the LS crisis management team, receive legal advice from peers trained as paralegals, and be referred to appropriate services.

# REFERENCES

1. Odek WO, Githuka GN, Avery L, et al. Estimating the size of the female sex worker population in Kenya to inform HIV prevention programming. *PLoS One* 2014;9(3):e89180. doi: 10.1371/journal.pone.0089180

2. Micro-Planning in Peer Led Outreach Programs - A Handbook. New Delhi: Bill & Melinda Gates Foundation, 2013.

3. National AIDS & STI Control Programme. Algorithm for managing common STI syndromes. Nairobi: National AIDS and STI Control Programme, 2015.

4. Bhattacharjee P, McClarty LM, Musyoki H, et al. Monitoring HIV Prevention Programme Outcomes among Key Populations in Kenya: Findings from a National Survey. *PLoS One* 2015;10(8):e0137007. doi: 10.1371/journal.pone.0137007

5. Decker MR, Crago AL, Chu SK, et al. Human rights violations against sex workers: burden and effect on HIV. *Lancet* 2015;385(9963):186-99. doi: 10.1016/S0140-6736(14)60800-X

6. Garcia J, Parker C, Parker RG, et al. "You're Really Gonna Kick Us All Out?" Sustaining Safe Spaces for Community-Based HIV Prevention and Control among Black Men Who Have Sex with Men. *PLoS One* 2015;10(10):e0141326. doi: 10.1371/journal.pone.0141326

7. Bhattacharjee P, Isac S, McClarty LM, et al. Strategies for reducing police arrest in the context of an HIV prevention programme for female sex workers: evidence from structural interventions in Karnataka, South India. *Journal of the International AIDS Society* 2016;19(4 Suppl 3):20856. doi: 10.7448/IAS.19.4.20856

8. FIDA Kenya. Documenting human rights violation of sex workers in Kenya: A study conducted in Nairobi, Kisumu, Busia, Nanyuki, Mombasa and Malindi, 2008.

9. Shields A. Criminalizing condoms: How policing practices put sex workers and HIV services at risk in Kenya, Namibia, Russia, South Africa, the United States, and Zimbabwe, 2012.
