# Supplementary material for: Key Programme Science lessons from an HIV prevention ‘Learning Site’ for sex workers in Mombasa, Kenya
Source: Sex Transm Infect. 2017 Dec 14;94(5):346–52. doi: 10.1136/sextrans-2017-053228 (PMC6204943; doi:10.1136/sextrans-2017-053228)
Supplement: Supplementary file 2 [file sextrans-2017-053228supp002.pdf]

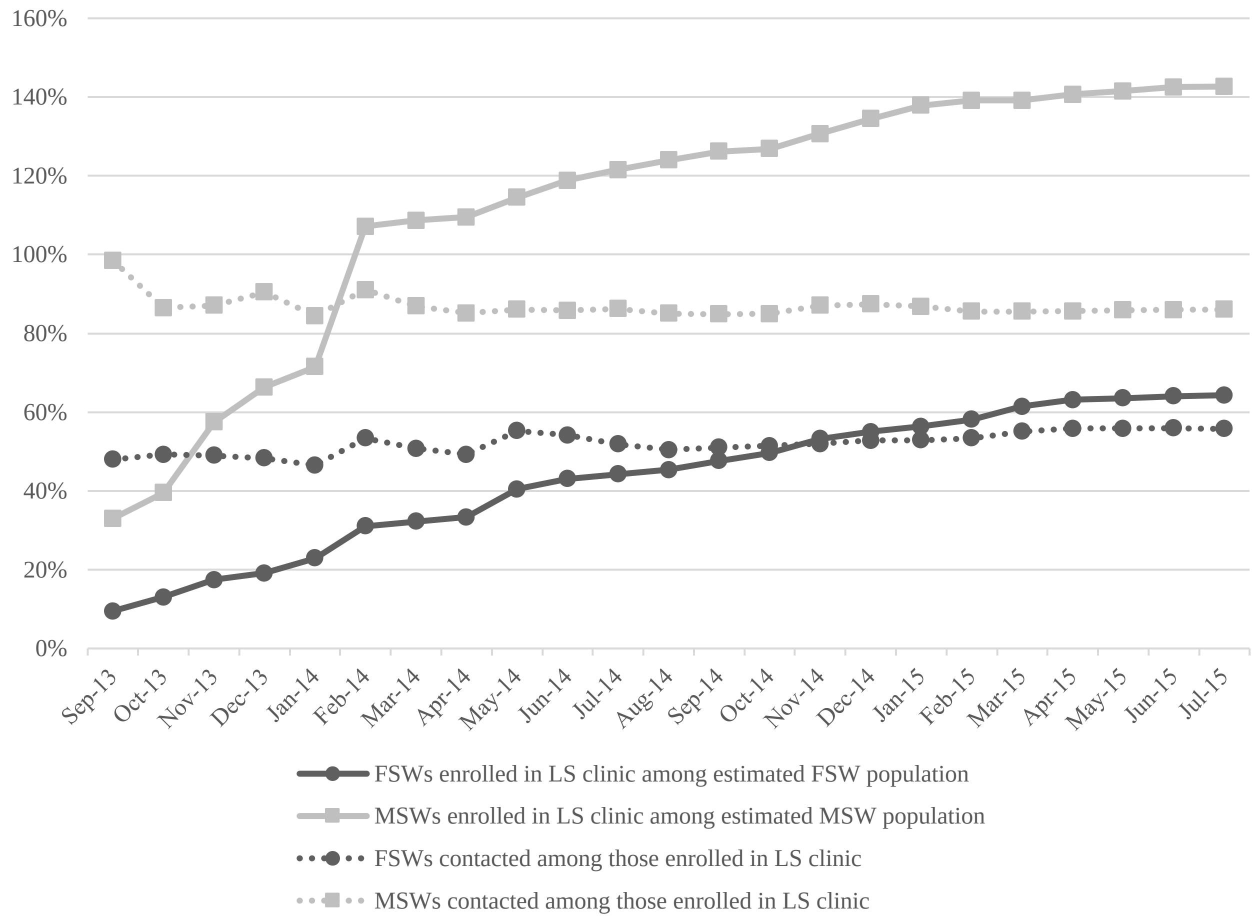

Web figure 1. Cumulative, monthly enrolment with the Learning Site clinic among estimated sex worker populations, and proportion of outreach-enrolled sex workers enrolling in the Learning Site clinic per month, September 2013 through July 2015.

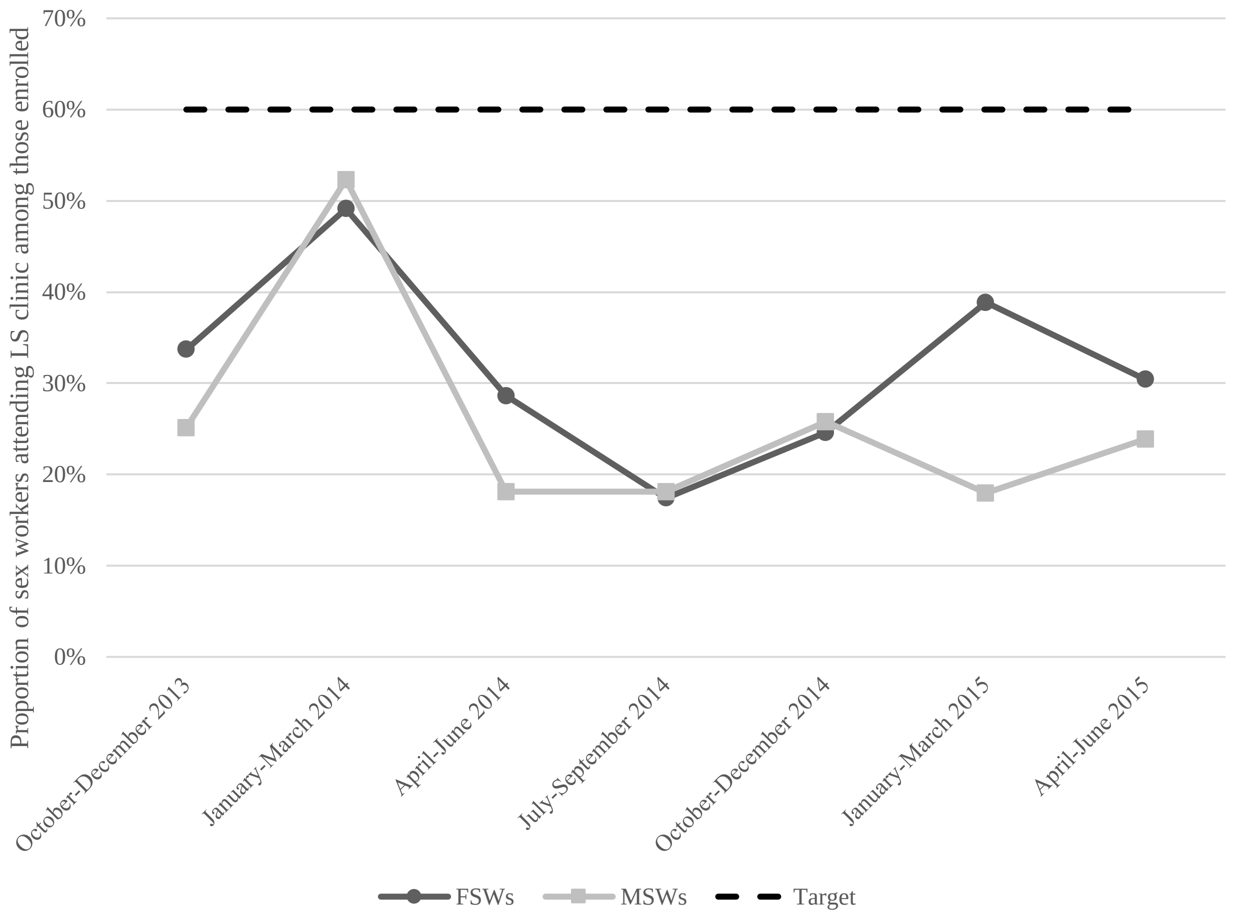

Web figure 2. Quarterly clinic attendance among sex workers enrolled with the Learning Site clinic, October 2013 through June 2015.

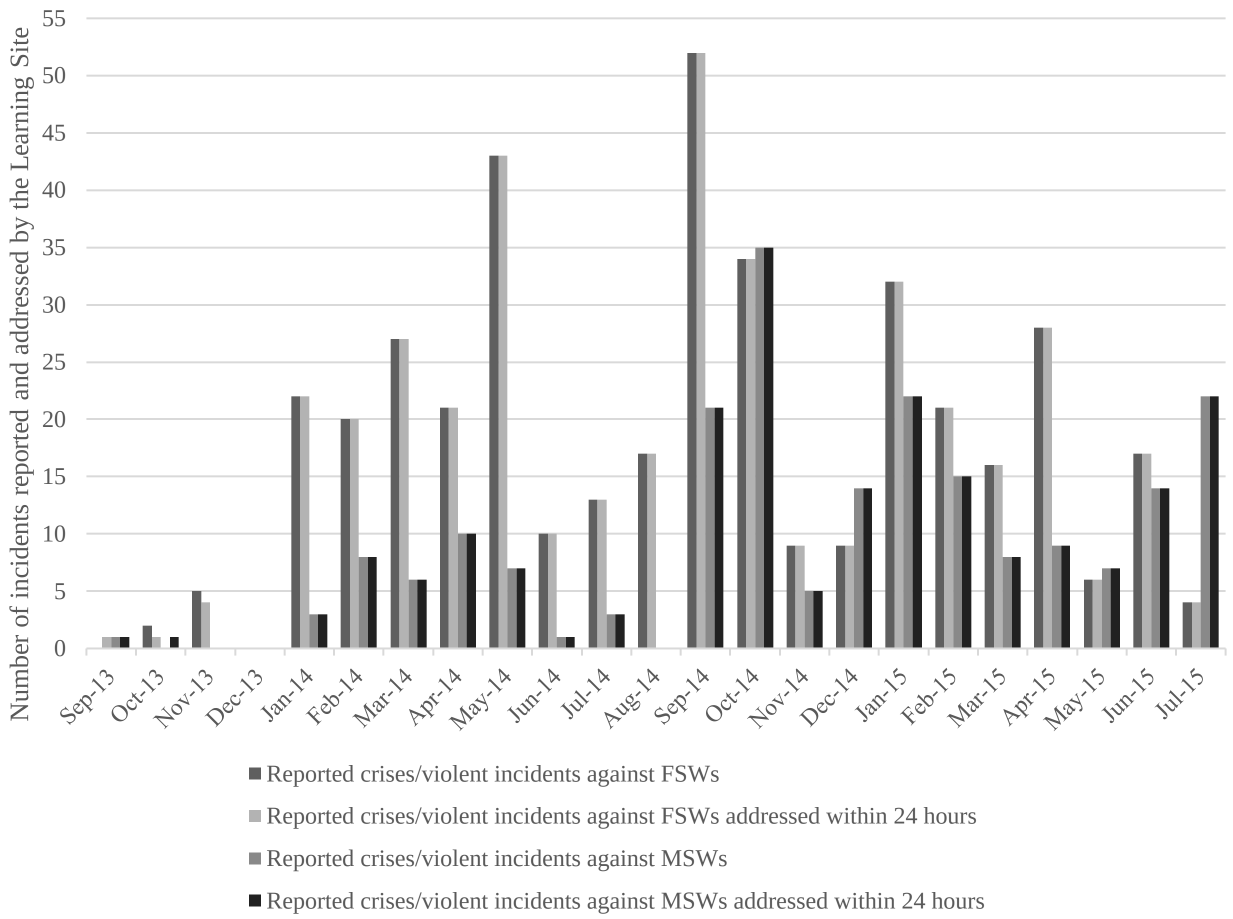

Web figure 3. Reports of and response to violent incidents among sex workers, and timely responses to violence, following sensitisation programmes organised by the Mombasa Learning Site, September 2013 through July 2015.
